# Registration of Midwives

**Published:** 1899-03-18

**Authors:** 


### Editor's Letter-Box.

**[Our Correspondents are reminded that prolixity is a great bar to publication, and that brevity of style and conciseness of statement greatly facilitate early insertion.]**

### REGISTRATION OF MIDWIVES.

The following letter has been addressed to the Chairman of the Midwives Bill Committee by the Council of the Incorporated Midwives' Institute, through Miss Rosalind Paget, hon. treasurer of the Midwives' Institute:—

"The Council of the Incorporated Midwives' Institute, after a conference with their members, have requested me, as their representative, to lay before your Committee their views on the present Bill.

"The Council of the Incorporated Midwives' Institute consider this Bill inferior to that of last year, but they are, however, so strongly impressed with the urgent need for legislation that they will offer no opposition to the present Bill so long as the following points are insisted on:—

"1. That the final decision in all cases be in the hands of the central authority. (See Clause 9, 'Duties of supervising authority'; and Clause 10, 'Annual and local certificate.') They consider with regard to Clause 10 that their interests are not sufficiently safeguarded.

"2. That their representation on the Central Board is retained, and that an amendment is asked for—'That the nominees of the Privy Council should be two lay persons, one a woman.'

"3. That the certificate of the London Obstetrical Society be retained as a qualification for licence.

"Your Committee will doubtless consider that these three points are some of them already dealt with in the Bill, and that the others will follow as a natural sequence. The reason the Council of the Institute have called your attention to them is because of the amendments to this Bill that have appeared in the *British Medical Journal*, and which will doubtless be largely supported by the medical profession.

"The Council of the Incorporated Midwives' Institute consider the machinery of the new clauses to be so complicated that they will prove unworkable, and provide only a 'nominal' protection to the mother, and the 'pretence' of a monopoly of practice to the licensed midwife. They consider the unconscientious midwife will have every opportunity of evading the law, while the conscientious midwife is controlled at every turn, and might possibly fall a victim to local jealousy owing to the preponderance of medical representation on the Midwives' Board. A further danger is created by the uncertainty regarding the official position of the person to be appointed as local supervising authority."

